# Generation of cattle knockout for galactose‐α1,3‐galactose and N‐glycolylneuraminic acid antigens

**DOI:** 10.1111/xen.12524

**Published:** 2019-05-22

**Authors:** Andrea Perota, Irina Lagutina, Roberto Duchi, Elisa Zanfrini, Giovanna Lazzari, Jean Paul Judor, Sophie Conchon, Jean Marie Bach, Tomaso Bottio, Gino Gerosa, Cristina Costa, Manuel Galiñanes, Jean Christian Roussel, Vered Padler‐Karavani, Emanuele Cozzi, Jean Paul Soulillou, Cesare Galli

**Affiliations:** ^1^ Avantea Laboratory of Reproductive Technologies Cremona Italy; ^2^ Fondazione Avantea Cremona Italy; ^3^ Centre de Recherche en Transplantation et Immunologie UMR 1064 INSERM, Université de Nantes Nantes France; ^4^ Institut de Transplantation Urologie Néphrologie (ITUN) CHU Nantes Nantes France; ^5^ IECM, Immuno‐endocrinology, EA4644 Oniris University of Nantes, USC1383 INRA, Oniris Nantes France; ^6^ Cardiac Surgery Unit ‐ Department of Cardiac Thoracic and Vascular Sciences and Public Health ‐ Padova University School of Medicine and CORIS Padova Italy; ^7^ Infectious Diseases and Transplantation Division, Institut d’Investigació Biomèdica de Bellvitge (IDIBELL) Hospitalet de Llobregat Barcelona Spain; ^8^ Reparative Therapy of the Heart, Vall d’Hebron Research Institute (VHIR) and Department of Cardiac Surgery University Hospital Vall d’Hebron, Autonomous University of Barcelona (AUB) Barcelona Spain; ^9^ Department of Thoracic and CardioVascular Surgery Nantes Hospital University Nantes France; ^10^ The George S. Wise Faculty of Life Sciences, Department of Cell Research and Immunology Tel Aviv University Tel Aviv Israel; ^11^ Transplant Immunology Unit Padua General Hospital Padua Italy

**Keywords:** bioprosthetic Heart Valve (BHV), cattle, *CMAH*, *GGTA1*, knockout, Neu5Gc, xenotransplantation, αGal

## Abstract

Two well‐characterized carbohydrate epitopes are absent in humans but present in other mammals. These are galactose‐α1,3‐galactose (αGal) and *N*‐glycolylneuraminic acid (Neu5Gc) which are introduced by the activities of two enzymes including α(1,3) galactosyltransferase (encoded by the *GGTA1* gene) and CMP‐Neu5Gc hydroxylase (encoded by the *CMAH* gene) that are inactive in humans but present in cattle. Hence, bovine‐derived products are antigenic in humans who receive bioprosthetic heart valves (BHVs) or those that suffer from red meat syndrome. Using programmable nucleases, we disrupted (knockout, KO) *GGTA1* and *CMAH* genes encoding for the enzymes that catalyse the synthesis of αGal and Neu5Gc, respectively, in both male and female bovine fibroblasts. The KO in clonally selected fibroblasts was detected by polymerase chain reaction (PCR) and confirmed by Sanger sequencing. Selected fibroblasts colonies were used for somatic cell nuclear transfer (SCNT) to produce cloned embryos that were implanted in surrogate recipient heifers. Fifty‐three embryos were implanted in 33 recipients heifers; 3 pregnancies were carried to term and delivered 3 live calves. Primary cell cultures were established from the 3 calves and following molecular analyses confirmed the genetic deletions. FACS analysis showed the double‐KO phenotype for both antigens confirming the mutated genotypes. Availability of such cattle double‐KO model lacking both αGal and Neu5Gc offers a unique opportunity to study the functionality of BHV manufactured with tissues of potentially lower immunogenicity, as well as a possible new clinical approaches to help patients with red meat allergy syndrome due to the presence of these xenoantigens in the diet.

## 1 INTRODUCTION

Two well‐characterized antigens are absent in humans but present in mammals and include galactose‐α1,3‐galactose (αGal) and *N*‐glycolylneuraminic acid (Neu5Gc) whose synthesis are catalysed by α(1,3) galactosyltransferase (encoded by the *GGTA1* gene)^1^, ^2^ and CMP‐Neu5Gc hydroxylase (encoded by the *CMAH* gene)^3^, ^4^, ^5^ respectively. These have been identified as major antigens in xenotransplantation studies or retrospective clinical findings^3^. Pigs that carry mutations in both genes, and therefore lack these xenoantigens, have been generated.^6^ Moreover, porcine kidneys lacking αGal are not hyperacutely rejected.^7^ It is also expected that such tissues will be less immunogenic for patients being implanted with animal‐derived tissues engineered to lack both antigens.

One of the major clinical applications of xenogenic tissues is for the manufacturing of bioprosthetic heart valves (BHVs), and it has been shown that such tissues carry the same xenoantigens despite the glutaraldehyde treatments used in the manufacturing process^8^, ^9^. Almost 300 000 patients are now undergoing BHV replacement each year^10^ with a growing demand. The sources of BHV are those manufactured from pig or bovine pericardia as compared to mechanical heart valve (MHV) that require lifelong anticoagulation therapy.

Bovine BHVs suffer however premature structural valve degeneration (SVD). The functionality of BHV is maintained for 10‐15 years in older patients. However, in younger (<35 years old) patients, BHVs undergo SVD much earlier.^11^ It is hypothesized that among various metabolic causes, SVD is also immune‐mediated since both αGal^8^, ^12^ and Neu5Gc^9^, ^13^ are still present on the BHV used in the clinic.

After BHV replacement, there is an increase of anti‐αGal antibodies^14^, ^15^ and it has been reported in an experimental context that implantation of BHV from αGal‐knockout pigs into primates is associated with a reduced anti‐αGal immune response.^16^ Moreover, valves from αGal/Neu5Gc‐deficient pigs further reduce human IgM/IgG binding when compared to BHV from wild‐type pigs^17^. A similar situation is likely to occur whether bovine double knockout (DKO) tissue would be used. Seventy per cent of the BHV currently used in the clinic are in fact manufactured with bovine pericardia, that carries non‐negligible amounts of αGal^8^ and of Neu5Gc^9^ even after currently used manufacturing treatments.

Pig‐ and cattle‐derived products are also a major source of proteins for human consumption, and particularly, cattle are the major source of dairy products. Such products can become allergenic for some patients or infants consuming baby milk replacers. This allergy, known as the red meat allergy syndrome,^18^, ^19^ generally follows a tick bite inducing an isotype shift for IgE against αGal antigen. Neu5Gc is not synthesized by humans, but it can be incorporated through the diet and found in minute amounts in endothelial or epithelial cells of various tissues, likely contributing to inflammation‐related diseases.^20^, ^21^ Furthermore, cattle can be used as a “bioreactor” to produce bioactive molecules for nutraceuticals or biomedical use, including r‐human lactoferrin^22^ in bovine milk. However, the resulting product differs from the human one because of the different glycosylation pattern.^23^ Similarly, partially “humanized” antibodies^24^ produced in cattle for various purposes still display Neu5Gc epitopes^25^, ^26^ that might be the target of an immune response by the host with clinically relevant side effects.

The scope of the present work was to generate cattle KO for both αGal and Neu5Gc antigens using a genome editing approach.^27^ A stillborn calf KO for αGal has been reported,^28^ but to the best of our knowledge, this work has not progressed further. Availability of DKO cattle line offers the opportunity to explore the potential of such animals to provide low immunogenic cattle‐derived products for clinical purposes as well as for the food industry and human consumption.

## 2 MATERIALS AND METHODS

### 2.1 Animal experiments and source of animals

All procedures involving the use of animals in this study were approved by the Animal Welfare Committee of Avantea and carried out in accordance with the Italian Law (D.Lgs 26/2014) and EU directive 2010/63/EU regulating animal experimentation after authorization by relevant authorities (Ministry of Health project n 991/2017‐PR). Bovine adult fibroblasts (BAFs) were derived from a skin biopsy of a Holstein bull and a cow with previous successful record of somatic cell nuclear transfer (SCNT). Recipient heifers used as surrogate mothers were also of Holstein breed.

### 2.2 Chemicals

All chemicals were purchased from Sigma‐Aldrich (Milano, Italy) unless otherwise stated.

### 2.3 PCR set‐up for identification and validation of target genes

Ensemble database was analysed to obtain the Reference genome sequences for the *GGTA1* (ENSBTAG00000012090) and of the *CMAH* (ENSBTAG00000003892) genes. These sequences were studied in silico to identify possible target sequences, and the selected regions were amplified by PCR and analysed with Sanger sequencing to exclude polymorphisms in male and female fibroblast cell lines selected for the genome editing.

Editing of *GGTA1* gene started initially in the male line targeting the exon 9 and because of the paucity of tools available at the time we never found efficient RNA guide. Therefore, we decided to use two guides that targeted the same sequence (Table 2, bt*GGTA1*cr1 and bt*GGTA1*cr2). Years later, when we targeted the female line, we were able to find an efficient guide for exon 4 (Table 3, bt*GGTA1*cr3) used for the pig by Sato et al.^29^ Editing of the *CMAH* gene was achieved efficiently in the exon 2 carrying the ATG codon. For the editing of the male, we used one guide (Table 2, bt*CMAH*cr1) and subsequently for the female we found a more efficient guide (Table 3, bt*CMAH*cr2).

Target exons and primers used for PCR analyses and Sanger sequencing of each gene are summarized in Table 1. All the synthetized oligonucleotides and the Sanger sequencing services were purchased from Eurofins Genomics, unless otherwise stated.

**Table 1:** Primers used for genotyping of bovine wild‐type cell lines, edited colonies and cloned animals

Oligo	Sequence (5′‐3′)	Gene	Target exon	Amplicon (bp)
FW1	GGATGCCTTTGATAGAGTTGG	*GGTA1*	9	440
RV1	GCTTTCATCATGCCATTGG			
FW2	AGCATCTTTCACAACTCAGG	*GGTA1*	4	739
RV2	TGAGACATTAGGAACATGGC			
FW3	TCAGGAGGAGACATCACCAACGG	*CMAH*	2	225
RV3	TGCCCATCCTACTTGTCGAGGG			

### 2.4 Genomic DNA extraction and PCR conditions

Primary fibroblasts and tissues biopsies were lysed at 55°C for 3 hours using a lysis buffer (100 mmol/L Tris HCl pH 8.3, 5 mmol/L EDTA pH 8.1, 0.2% SDS, 200 mmol/L NaCl) supplemented with Proteinase K (300 µg/mL; Macherey‐Nagel). Genomic DNA was extracted (Sambrook et al, 1989) and resuspended with TE buffer. All the amplifications were performed using Takara La Taq DNA Polymerase (Takara, Japan).

Polymerase chain reaction conditions for *GGTA1* in the male line (exon 9) were as follows (FW1 + RV1 = 440 bp): 94°C, 2 minutes; 94°C, 30 seconds, 72°C (−1°C/cycle), 30 seconds; 72°C, 15 seconds for 8 cycles; 94°C, 30 seconds, 58°C, 30 seconds; 72°C, 15 seconds for 35 cycles; and a final extension step of 72°C for 7 minutes. PCR conditions for *GGTA1* in the exon 4 of the female line were as follows (FW2 + RV2 = 739 bp): 94°C, 2 minutes; 94°C, 30 seconds, 60°C, 30 seconds, 72°C, 30 seconds for 35 cycles; and a final extension step of 72°C for 7 minutes.

For *CMAH,* PCR conditions were as follows (FW3 + RV3 = 225 bp): 94°C, 2 minutes; 94°C, 30 seconds; 58°C, 30 seconds; 72°C, 30 seconds for 40 cycles; and a final extension step of 72°C for 7 minutes.

Determination of the absence of any genomic polymorphisms was achieved cloning each resulting PCR products in *E coli* using the TOPO TA cloning kit (Thermo Fisher Scientific). Resulting purified plasmids (Plasmid Mini kit, Qiagen) were subjected to Sanger sequencing analyses (Eurofins Genomics).

### 2.5 CRISPR/Cas9 plasmid constructs, single guide RNA synthesis and design of ss*CMAH*‐STOP oligonucleotide

Editing of the *GGTA1* and *CMAH* genes of the bovine male line was achieved by cloning and expressing the desired single guide RNAs (sgRNAs), into the pX330‐U6‐Chimeric_BB‐CBh‐hSpCas9 expression vector, that was a gift from Feng Zhang (Addgene plasmid # 42230). DNA oligonucleotides for sgRNAs (Table 2) were purchased from Eurofins Genomics. Annealing and molecular cloning of gene‐specific complementary oligos were done following the protocol described by Cong and colleagues.^30^ The resulting purified expression vectors (Plasmid mini kit, PC‐20, Qiagen) were verified by Sanger sequencing before transfection.

**Table 2:** Oligonucleotides synthetized for the assembly of desired CRISPR/Cas9 expression vectors used for the male line and sequence of the ss*CMAH*‐STOP oligo

Oligo	Sequence (5′‐3′)	Guide sequence—PAM (5′‐3′)	Target gene (exon)	Expression vector
bt*GGTA1*cr1 FW	CACCGGAGACCCTGGGCGAGTCGG	GGAGACCCTGGGCGAGTCGG‐TGG	*GGTA1* (9)	pX330‐bt*GGTA1*cr1
bt*GGTA1*cr1 RV	AAACCCGACTCGCCCAGGGTCTCC			
bt*GGTA1*cr2 FW	CACCGCTGGGCCACCGACTCGCCC	GCTGGGCCACCGACTCGCCC‐AGG	*GGTA1* (9)	pX330‐bt*GGTA1*cr2
bt*GGTA1*cr2 RV	AAACGGGCGAGTCGGTGGCCCAGC			
bt*CMAH*cr1FW	CACCGACTATGGGCAGGCAAGTGA	GACTATGGGCAGGCAAGTGA‐GGG	*CMAH* (2)	pX330‐bt*CMAH*cr1
bt*CMAH*cr1 RV	AAACTCACTTGCCTGCCCATAGTC			
ss*CMAH*‐STOP oligo	GTGACAGCTGCCATTCTTCTGAAATACCCAGGGAGAGGCAACGACAGACTTAAGGCAGGCAAGTGAGGGAGGCATTACTTTGCTGGGAAGGTGGGGTCAA	//	//	//

Edited female colonies were obtained transfecting the Cas9 protein/gRNA ribonucleoprotein complexes (Cas9‐RNPs).^31^, ^32^ Desired sgRNAs (bt*GGTA1*cr3 and bt*CMAH*cr2) were in vitro synthetized following the CRISPOR guidelines (http://crispor.org/). Briefly, oligonucleotides (Table 3) were annealed, amplified and purified before to use the resulting amplification product as template (1μg) for the following transcription step. Single guide RNAs were finally synthetized using the TranscriptAid T7 High Yield Transcription kit (Thermo Fisher Scientific) and purified on silica membranes columns (MEGAclear Transcription clean‐up kit, Thermo Fisher Scientific) according to the manufacturer's instructions and stored at −80°C.

**Table 3:** Oligonucleotides used for in vitro T7 transcription of sgRNAs used for the female line

Oligo	Sequence (5′‐3′)	Guide sequence—PAM (5′‐3′)	Target gene (exon)
bt*GGTA1*cr3sgRNA	GAAATTAATACGACTCACTATAGAGAAAATAATGAATGTCAAGTTTTAGAGCTAGAAATAGCAAG	GAGAAAATAATGAATGTCAA‐AGG	*GGTA1* (4)
bt*CMAH*cr2sgRNA	GAAATTAATACGACTCACTATAGAGAGGCAACGACAGACTATGTTTTAGAGCTAGAAATAGCAAG	GAGAGGCAACGACAGACTAT‐GGG	*CMAH* (2)
sgRNAT7common	AAAAGCACCGACTCGGTGCCACTTTTTCAAGTTGATAACGGACTAGCCTTATTTTAACTTGCTATTTCTAGCTCTAAAAC	//	//

**Figure 1:**
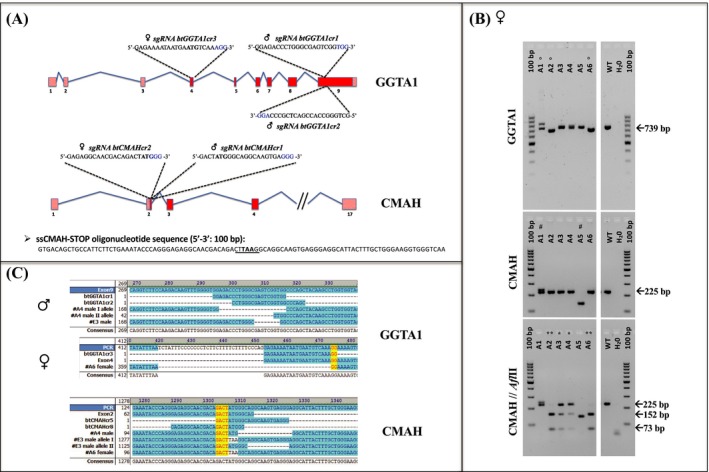
Editing of *GGTA1* and *CMAH* genes in male and female fibroblasts. A, Target sequences for selected sgRNAs and ss*CMAH*‐STOP oligo sequence. For each bovine gene (*GGTA1* and *CMAH*), target sequences are indicated on the respective exons recognized by the selected sgRNAs. PAM sequences are highlighted in blue. In the ss*CMAH*‐STOP oligo sequence, the TAA (STOP) codon is highlighted in bold character; the *Afl*II restriction site is underlined. B, PCR analyses of female colonies. The results of the PCR analyses performed for the genomic characterization of the female colonies (A1, A2, A3, A4, A5 and A6) selected after Dynabeads sorting are reported as an example. Each colony was analysed for the *GGTA1* gene (739 bp) and for the *CMAH* gene (225 bp). Resulting electrophoretic patterns determined directly that some colonies were characterized by visible *Indels*, creating bands different from the WT controls. This situation is clear for colonies A1 (double band), A2 (deletion) and A6 (deletion) in PCR analyses for the *GGTA1* gene (°) and for colonies A1 (double band) and A5 (deletion) in PCR analyses for the *CMAH* gene (#). Resulting *CMAH*‐PCR products were also digested with the *Afl*II restriction enzyme, detecting the alleles interested by the targeting event. Due to the introduction of a STOP codon (TAA) in the START position (ATG) of the CMAH gene, only the HDR‐*CMAH* alleles will be cut by the restriction enzyme producing two lower bands (152 + 73 bp). A simple agarose electrophoresis enabled us to identify possible additional edited colonies detecting the STOP codon insertion (**) for colonies A2 and A6 and the single insertion (*) for colonies A3 and A4. In these last ones, the not targeted allele resulted uncut (225 bp) as the WT sample. For this reason, the final determination of the exact *Indels*, occurred in all the edited colonies, was determined by Sanger sequencing of the resulting TOPO TA *E coli* clones. 100 = 100 bp ladder (Thermo Fisher Scientific); A1, A2, A3, A4, A5 and A6 = transfected females colonies; WT = wild‐type female line; H_2_0 = Nucleases‐free water. C, Sequences alignments of colonies used for the SCNT. Sanger sequencing outlining the mutations affecting the *GGTA1* and the *CMAH* genes of colonies selected for the SCNT step. For the *GGTA1* gene, the exon 9 was used as reference for the male colonies and a PCR product including the exon 4 was used for the female ones. In both cases, deletions of different lengths were obtained (Table S1). For the CMAH gene, all edited alleles of the edited colonies were aligned using as reference a PCR product including the exon 2 sequence. In this case, in both lines, we were able to determine the TAA substitution, as result of the targeting event mediated by the site‐specific cut, produced by the CRISPR/Cas9 system driven by the sgRNA bt*CMAH*cr1

We targeted the C*MAH* gene using as template a synthetized single strand oligonucleotide (ss*CMAH*‐STOP oligo) specific for the exon 2 and symmetric according to the position of the *CMAH*‐START codon. Its sequence is characterized by the substitution of the START codon (ATG) with a STOP codon (TAA, in bold Figure 1A), generating a new *Afl*II restriction site (CT **TAA** G, underlined in Figure 1A), useful for the identification of the knock‐in colonies (152bp + 73 bp) with the *Afl*II‐RFLP analyses (*Afl*II from Thermo Fisher Scientific; 1 hour at 37°C).

### 2.6 Culture, transfection and selection of adult fibroblasts

Bovine adult fibroblasts (male and female) were cultured in DMEM + M199 (1:1) +10%FCS in 5%CO_2_, 5%O_2_ at 38°C.

Male fibroblasts (2 × 10^6^ cells) were transfected using Nucleofector (V‐024 program, Lonza), two µg of each the 3 CRISPR/Cas9 expressing vectors (pX330‐bt*GGTA1*cr1 and pX330‐bt*GGTA1*cr2—exon9 of *GGTA*1 gene; pX330‐bt*CMAH*cr1—exon 2 of *CMAH* gene) and 0.4 nmol of the ss*CMAH*‐STOP oligo (IDT).

Female fibroblasts (1 × 10^6^ cells) were transfected using Neon system (P‐9 program; Thermo Fisher Scientific) and the Cas9‐RNP complex format of the *S pyogenes.* Cas9‐RNP was obtained mixing the recombinant Cas9 protein (14.4 μg; Edit‐R Cas9, Dharmacon), with the bt*GGTA1*cr3‐sgRNA (3.6 μg), the bt*CMAH*cr2‐sgRNA (3.6 μg) and 0.4 nmol of the ss*CMAH*‐STOP oligo.

Transfected cells were plated in a 60‐mm dish and cultured for 3 days when they were passaged 1‐3. Day 7 male (6.5 × 10^6^ cells) and D5 female (2.3 × 10^6^ cells) αGal‐negative cells were selected using biotin‐conjugated IB4 lectin attached to streptavidin‐coated magnetic beads.^33^, ^34^, ^35^ Cells were harvested (6.5 × 10^6^ cells) and suspended in 0.2 mL PBS containing 1 µg biotin‐conjugated IB4 lectin (Sigma) and 0.1 mg Dynabeads M‐280 streptavidin (Life Technologies). The αGal‐positive cells were removed using a magnetic rack. The procedure was repeated three times. The αGal‐negative cells were plated on 150‐mm plates and cultured for 9‐10 days when the largest colonies with good morphology were picked up and expanded.

For each colony, one aliquot was cryopreserved in liquid nitrogen (DMEM/TCM199 1:1, 20% FBS and 10% DMSO) for subsequent SCNT and another was lysed for DNA extraction and molecular analyses (PCR, *Afl*II‐RFLP, TOPO TA cloning and Sanger sequencing).

Only colonies that, during the *CMAH* molecular screenings, presented detectable *Indels* in their PCR products and/or that resulted positive for the *Afl*II‐RFLP assay (152 bp + 73 bp) were subjected to the Sanger sequencing analyses for both genes (*GGTA1* and *CMAH*), detecting the occurred *Indels* and the successful ss*CMAH*‐STOP oligonucleotide knock‐in events.

Eight (four male and four female) confirmed DKO colonies, edited for *GGTA1* and *CMAH* genes, were selected for further screening in SCNT to assess developmental potential. Before SCNT embryos were transferred into recipients, at least 10 cloned embryos of each selected colony were analysed for the absence of wild‐type genotypes. Genomic DNA extraction procedure, PCR amplification and sequencing reactions were done using the same materials and methods described above.

### 2.7 Somatic cell nuclear transfer (SCNT)

The protocol used is described in Galli et al^36^ with minor modifications. Briefly, the bovine ovaries were collected at a local abattoir. Follicles larger than 3 mm were aspirated, and cumulus‐oocyte complexes were selected and in vitro matured in TCM 199 supplemented with 10% (v/v) foetal calf serum, 1 mg/mL 17 b‐oestradiol, ITS, 100 mg/mL sodium pyruvate, 90 mg/mL L‐cysteine, 720 mg/mL glycine, 7 nL/mL b‐mercaptoethanol, gonadotropins (0.05 IU/mL FSH and 0.05 IU/mL LH; Meropur 75, Ferring) and growth factors (50 ng/mL long‐EGF and 10 ng/mL bFGF) at 38.5°C in 5% CO_2_ in humidified air for 22 hours. The day before SCNT, nuclear donor cells were induced into quiescence by serum starvation (0.5% FCS). The day of SCNT, cells were trypsinized and resuspended in H‐SOF^37^ buffered with 25 mmol/L Hepes (H‐SOF) used for all manipulations. Oocytes with an extruded polar body were stained with Hoechst 33342 (5 μg/mL) and enucleated in the presence of cytochalasin B (5 μg/mL) by the aspiration of polar body and associated metaphase II plate in minimal volume of ooplasm under UV. Donor cells were transferred in the perivitelline space of enucleated oocytes. Donor cell‐cytoplast couplets were washed in 0.3 M mannitol solution and fused by double DC‐pulse (1.5 Kv/cm) 30 µsec long and returned into maturation medium. After one hour, at about 27‐29 hours of maturation, NT embryos were activated with 5 µmol/L ionomycin for 4 minutes followed by 3 hours of incubation in mSOF medium supplemented with 1 mmol/L 6‐DMAP and 5 μg/mL cycloheximide. At the end of the activation, reconstructed embryos were cultured in mSOF supplemented with essential and non‐essential amino acids and 4 mg/mL BSA up to 7‐8 days to the blastocyst stage.

### 2.8 Recipients synchronization, embryo transfer (ET) and calving

Heifers of 14‐16 months of age were used as recipients. Oestrus was synchronized using the Ovsynch protocol with two injections of a GnRH analogue (Dalmarelin, Fatro, Italy) 8 days apart. Forty‐eight hours after the second injection, animals were observed for oestrus signs and 6 days later those that showed oestrus were ultrasound scanned to detect the presence of a corpus luteum (CL). Those that had a well‐developed CL received 1 or 2 embryos (either fresh or frozen thawed) by non‐surgical ET ipsilateral to the CL. Four weeks after ET, pregnancy diagnosis was performed by ultrasound scanning and then the pregnant animals were checked at monthly interval till the end of the pregnancy. The delivery of the calves was by elective caesarean section at 280 days of gestation.

### 2.9 Genotyping and phenotyping analyses for αGal and Neu5Gc antigens in DKO cattle‐derived primary cells

Newborn calves were subjected to ear biopsy to establish a primary cell line, to extract the genomic DNA for genotyping by PCR and DNA sequencing as described above. Resulting primary fibroblasts for each calf were expanded and cryopreserved in DMEM:TCM199 1:1 with 10% DMSO and 20% FCS in CBS straws (IMV, Italy). For FACS analysis, bovine fibroblasts were thawed and cultured in DMEM medium (Gibco), with 10% FBS, 1% Peni‐Strepto and bFGF (Sigma, 1 ng/mL). Once confluent, cells were trypsinized and split into two culture dishes, one with the complete medium as above and the other with DMEM medium, 5% human serum (Sigma), 1% Peni/strepto and bFGF (1 ng/mL). For αGal analysis, cells were trypsinized, resuspended and washed in PBS + BSA 0.1%. The cells were pelleted (750*g* x 1 minute, 4°C) and resuspended in PBS + BSA 0.1% containing the FITC coupled lectin (BS‐I Isolectin B4) diluted 1:50 and incubated at 4°C for 30 minutes. After 3 washes in PBS BSA 0.1%, cells were ready for FACS analysis.

For Neu5Gc analysis, cells were cultured for at least 2 weeks in DMEM + human serum. Foetal calf serum is rich in Neu5Gc that is incorporated by cells in culture. Therefore, to avoid false positives, the cells used for the FACS analysis have to be cultured for at least 2 weeks in culture media without Neu5GC, by replacing FCS with human serum. Cells were seeded in 96‐well plates (10^6^ cells/well), washed once with PBS with 0.5% fish gelatin (PBS‐FG) and then incubated in 200 µL PBS‐FG with anti‐Neu5Gc antibody or control isotype (BioLegend, chicken polyclonal IgY, dilution 1 :1000) for 1 hour at 4°C, washed four times in PBS‐FG, incubated in 100 µL PBS‐FG with Alexa 647‐coupled anti‐IgY antibody (Jackson ImmunoResearch, F(ab′)_2_ fragment donkey anti‐chicken 1:500) for 1 hour at 4°C, washed four times in PBS‐FG and transferred into FACS tubes. FACS analysis was conducted using a BD Pharmingen LSR‐II flow cytometer and FlowJo software (TreeStar). Despite this culture period, where the cells are also not growing under optimal conditions, sometimes it is not sufficient to clear all the carry‐over of Neu5Gc due to culture conditions and often some background staining remains like the one observed in Figure 4.

## 3 RESULTS

### 3.1 Disruption of *GGTA1* and *CMAH* genes in primary bovine fibroblast lines

Two millions male fibroblasts were nucleofected and expanded for 7 days to 6.5 × 10^6^. After Dynabeads sorting, 4200 αGal‐negative cells (0.065%) were recovered and plated in 20 Petri dishes (∅ = 150 mm) for clonal selection. Ten days after plating, 41 (1%) best growing colonies were picked up for PCR analysis and SCNT. Editing of the female fibroblasts took place a year later, and we used a different system using neon transfection with the Cas9 protein for the first time. From the one million female fibroblasts transfected with Neon and the Cas9‐RNP at D5, 2.3 × 10^6 ^cells were used for Dynabeads sorting. The efficiency of transfection was very low compare to the editing of male fibroblasts that was obtained using a plasmid for transfection but all αGal‐negative cells were plated in one 150‐mm dish and after 9 days 6 colonies were picked up. Of the 41 male colonies selected by pick up and analysed, *CMAH*‐PCR and *Afl*II‐RFLP analyses revealed that 15 appeared to be edited for the CMAH gene and for this reason they were sent for Sanger sequencing analyses of their *GGTA1* and *CMAH* genes (Figure 1C and Table S1). *GGTA1*‐KO was confirmed in all 15 colonies and 13 (31.7%) resulted also KO for Neu5Gc (Table 4).

**Table 4:** Fibroblasts colonies—screening results

Bovine line	Picked colonies	*GGTA1*‐KO (beads)	*CMAH*‐KO (PCR + RFLP)	*GGTA1*‐KO (sequencing)	*CMAH*‐KO (sequencing)
Male	41	41	15	15	13
Female	6	6	6	6	4

The female colonies were subjected to the same analysis. The PCR analysis (Figure 1B) followed by Sanger sequencing analyses (Figure 1C and Table S1) on both genes of the six female colonies revealed that two colonies were heterozygotes and four colonies were KO (66.6%) for the *CMAH* gene and that all six colonies were *GGTA1*‐KO (Table 4), confirming the high efficiency of Dynabeads selection for the *GGTA1* KO.

Male A4 and E3 and female A6 colonies were used for SCNT based on their morphology and growing characteristics and embryo production after SCNT. Ten SCNT embryos of each colony were sequenced to confirm the purity of the selected colonies for the required mutations (Figure 1C) to avoid potential contaminations of WT cells.

### 3.2 Generation of DKO calves by SCNT

**Figure 2:**
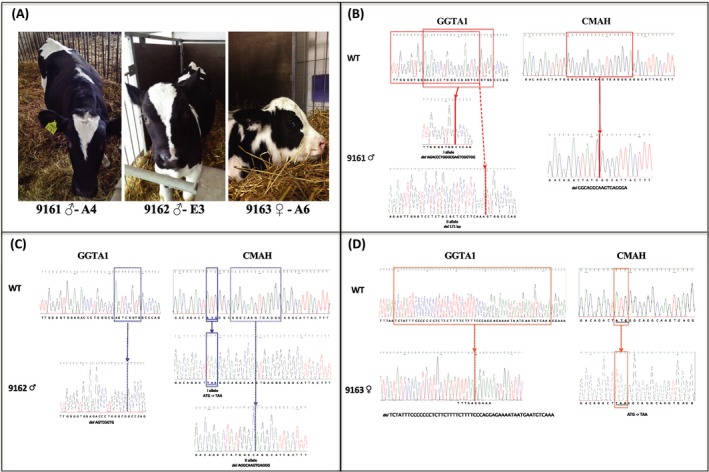
DKO calves and sequencing results. A, Pictures of cloned DKO calves. Two healthy cloned bull calves (9161 and 9162) were generated from two different DKO colonies (A4 and E3). Cloned heifer calf was generated using the colony A6. B, Sequencing results for 9161. For the *GGTA1* gene, it was confirmed that this gene is affected by two different deletions (21 and 171 bp), as previously described for the edited colony A4 (Table S1). These data were finally demonstrated by the deletion (17 bp) generated in the *CMAH* gene. C, Sequencing results for 9162. The *GGTA1* gene sequence presented a 8 bp deletion, and the CMAH gene is characterized by the same 2 different mutations (TAA substitution; del 13 bp) detected in colony E3 (Table S1). D, Sequencing results for 9163. The same *Indels,* characterizing the *GGTA1* (del 54 bp) and the *CMAH* (TAA substitution) genes of A6 colony (Table S1), were confirmed

Two male DKO colonies (A4 and E3) were used as nuclear donors for SCNT (Table 5). Seven blastocysts (BLs), derived from colony A4, were transferred in 5 synchronized recipients, 4 (80%) became pregnant and 1 pregnancy (25%) went to term delivering 1 calf (9161, Figure 2A). Fifteen BLs derived from colony E3 were transferred in 12 synchronized recipients; 5 (41.6%) became pregnant, 1 pregnancy (20%) went to term delivering 1 calf (9162, Figure 2A).

**Table 5:** Development of cloned embryos after transfer into recipient heifers

Bovine line	No. of colonies	No. of embryos	No. of recipients	No. of pregnant (%)	No. of born alive at term
Male	A4	7	5	4 (80)	1
Male	E3	15	12	5 (41.6)	1
Female	A6	31	16	6 (37.5)	1

Female DKO colony A6 was used for SCNT, and 31 BLs were transferred in 16 recipients; 6 (37.5%) became pregnant and 1 pregnancy went to term delivering 1 calf (9163, Figure 2A).

### 3.3 Genotyping of cloned calves

Sanger sequencing of TOPO TA‐cloned PCR products of DKO calves confirmed the *Indels* characterizing the colonies used for cloning. In details, in clone 9161, *GGTA1* gene is affected by two different mutations in exon 9 (del AGACCCTGGGCGAGTCGGTGG/del 171bp) and the exon 2 of the *CMAH* gene carries a deletion (del GGCAGGCAAGTGAGGGA) as it was described for colony A4 (Table S1; Figure 2B). In clone 9162 (Figure 2A), a deletion in *GGTA1* gene (del AGTCGGTG) is accompanied by 2 different mutations in the exon 2 of *CMAH* gene. The first allele was inactivated by the substitution of the ATG codon (START) with the TAA codon (STOP), due to the homology‐directed repair (HDR) event driven by the ss*CMAH*‐STOP oligo, as described for colony E3 (Table S1, Figure 2C), and the second allele has 13 bp deletion (del AGGCAAGTGAGGG).

Sanger sequencing results of female clone 9163 (Figure 2A) demonstrated that a deletion in the exon 4 of the *GGTA1* gene (del 54bp) and the substitution of the START to a STOP codon (ATG  TAA) in the exon 2 of the *CMAH* gene are identical to the *Indels* described for the donor female colony A6 (Table S1, Figure 2D). PCR analyses on the male calves (data not shown) demonstrated also that the CRISPR/Cas9 expression vectors were not integrated in the genome of the cloned calves.

### 3.4 Phenotyping of cloned calves

**Figure 3:**
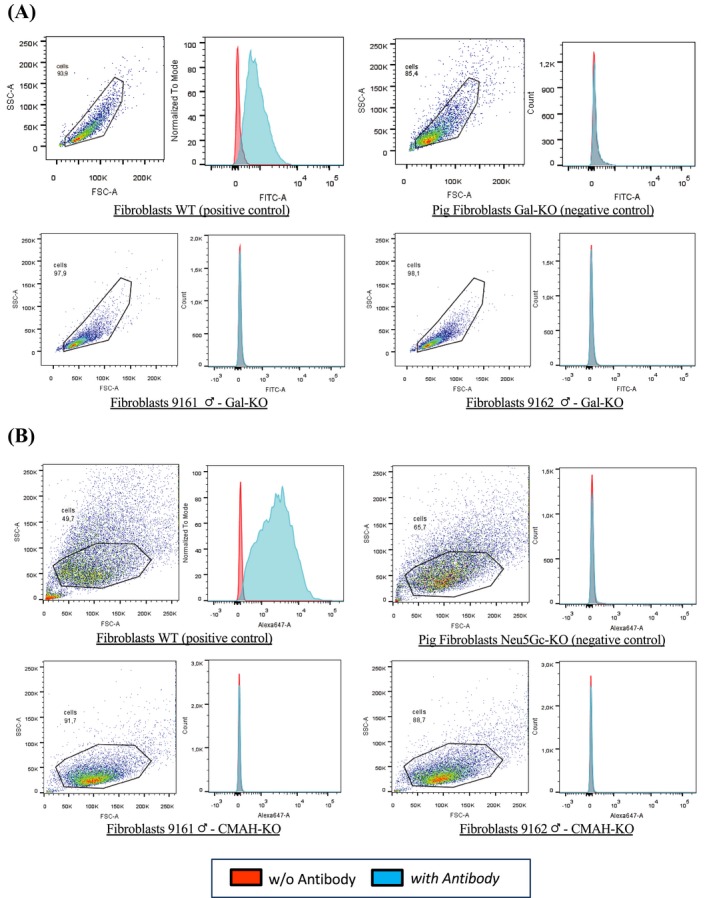
FACS analyses for 9161, 9162 male calves. Fibroblasts from wild‐type animal (WT) and from the edited male calves were analysed by FACS. As negative controls, pig DKO fibroblasts were used as no bovine material was available. The results demonstrated that the αGal (A) and (B) Neu5Gc antigens were absent from the cell surface of cloned calves, confirming the genotype analyses for the knocked‐out genes (*GGTA1* and *CMAH*). Fibroblasts WT (positive control): wild‐type primary fibroblasts from the bovine line prior to genetic modification expressing the αGal and the Neu5Gc antigens. Pig fibroblasts Gal‐KO and Neu5Gc‐KO (negative control): porcine primary fibroblasts NOT expressing the αGal and the Neu5Gc antigens. Fibroblasts 9161/9162 Gal‐KO and Neu5Gc‐KO: primary fibroblasts derived from cloned DKO calves

**Figure 4:**
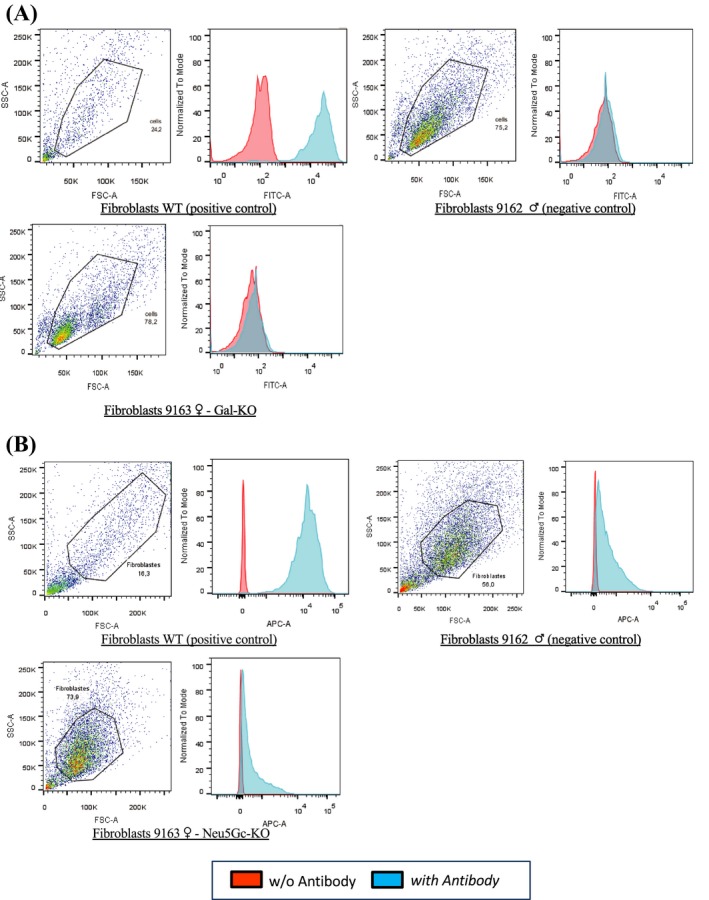
FACS analyses for DKO female 9163 calf. Fibroblasts from wild‐type animal (WT) and from the edited female calf were analysed by FACS. As negative controls, FACS‐validated DKO fibroblasts from 9162 male calf were used. The results demonstrated that the αGal (A) and (B) Neu5Gc antigens were absent from the cell surface of cloned female calf, confirming the genotype analyses for the knocked‐out genes (*GGTA1* and *CMAH*). Fibroblasts WT (positive control): wild‐type primary fibroblasts from the bovine line prior to genetic modification expressing the αGal and the Neu5Gc antigens. Fibroblasts 9162 Gal‐KO and Neu5Gc‐KO (negative control): bovine primary fibroblasts NOT expressing the αGal and the Neu5Gc antigens. Fibroblasts 9163 Gal‐KO and Neu5Gc‐KO: primary fibroblasts derived from cloned DKO female calf

FACS analysis confirmed the genotyping results of the three calves. All primary cell lines derived from biopsies of the cloned male calves do not express αGal (Figure 3A) and Neu5Gc (Figure 3B) as opposed to WT control cells before genetic engineering. As negative controls, pig cells KO for both antigens were used. The female phenotyping was performed in the same way as for the males but in this case the negative control was the male 9162. In this experiment performed a year later with different experimental context, the αGal was completely negative. In the case of Neu5Gc, both the control (9162) and the female (9163) had some background staining. Since the 9162 pictured in Figure 3 is the same as in Figure 4, we can conclude that the tail of Neu5Gc staining is background staining coming from the different experimental setting and culture conditions.

## 4 DISCUSSION

In this work, we targeted two well‐known xenoantigens identified as such from pig xenotransplantation studies that are also expressed in cattle. Here, we show that editing bovine fibroblasts are possible using both CRISPR/Cas9 in plasmid and Cas9‐RNP formats and live animals can be generated through SCNT. The advent of programmable nucleases for genome editing in large animals, especially the pig, has greatly increased its efficiency by reducing the number of animals required and the costs involved. The number of genetically modified pigs and the consequent generation of animal models through precise genetic engineering have grown exponentially in the last 10 years. However, genetically modified cattle are still very few due to some constrains for applying this technology to this species such as the long generation interval. Nevertheless, cattle would be more relevant for food production since it is a major source of beef and dairy products. Furthermore, one of the major potential applications for DKO cattle for both *GGTA1* and *CMAH* would be as a source of less immunogenic biological materials (pericadia) to manufacture BHV. In addition, genetically engineered cattle would also allow to produce food to avoid, for example, anaphylactic reaction following the consumption of red meat in some allergic individuals.

Despite the low transfection efficiency in bovine fibroblasts that affected the total number of edited colonies, because of the thorough screening of the few colonies selected and the combination with SCNT, we were able to generate DKO male and female calves. All the bovine genome editing work was undertaken to disrupt simultaneously the *GGTA1* and the *CMAH* genes without the need of a selectable marker, choosing primary cell lines whose genomic sequences were not affected by polymorphisms. We started the bovine genome editing work in the male line transfecting the plasmid format of the *S pyogenes* CRISPR/Cas9 system, while later its Cas9‐RNP format was tested in the female line.

We selected to target exon 9 of *GGTA1* gene in the male line using together two different sgRNAs (bt*GGTA1*cr1 and bt*GGTA1*cr2—Figure 1A). In contrast to the female line, we targeted the exon 4, using the protein (Cas9‐RNP), designing a sgRNA specific for the START codon (bt*GGTA1*cr3 Figure 1A). The use of Dynabeads and IB4 lectins greatly compensated for the low transfection efficiency very effectively since all the analysed colonies derived from cells that did not bind IB4 were all KO for the *GGTA1* (Table 4). As a consequence, also the KO rate for the CMAH gene was very high indicating that when these nucleases enter the cells, they are very effective on all the targets. This event was also described for the pig by Li et al.^38^ The use of ssODN‐mediated KI with CRISPR/Cas9 system for KO purposes was also possible in cattle and facilitated the PCR screening because of the insertion of an *Afl*II restriction site. The use of the plasmid to introduce and express all the machinery required was in our experiments more efficient than the use of the protein but because we required only a few cell clones for SCNT, it did not affect the success at the end since we had far more cell clone that we could need for SCNT. The reason for preferring the Cas9 protein to the plasmid is to avoid the risk of integration of the plasmid. Luckily in this case, we did not detect any integration of the CRISPR/Cas9 expressing plasmids in the genome of the male calves. CRISPR/Cas9‐mediated genome editing procedures are compatible with SCNT, and the efficiency is comparable when WT cells are used. Three live calves were delivered by caesarean section and the first born (9161) is about to reach puberty while the female is only a few days old. The genotype of the three claves born alive exactly matched the genotype of the three cell clones selected for SCNT (Table S1). To further validate the genotyping findings with the phenotype, FACS analysis was performed on fibroblasts derived from the three newborn animals. The absence of αGal and Neu5Gc was clearly confirmed on the two bull calves. To perform the FACS analysis, primary cells were grown from biopsy taken in the first days of life of the calves that were gestated by WT surrogate mother and fed after birth with milk from WT cows; moreover, the culture of primary cells was performed with FCS supplementation to culture media from WT source. All these conditions favour incorporation of Neu5Gc into the cells that before the analysis requires 2‐3 week in culture with serum lacking Neu5Gc. We used human serum in this period to allow the cells to shed the incorporated Neu5Gc but this time is variable depending on culture conditions, not ideal with human serum for bovine fibroblasts and the reagents used. The phenotypical characterization of the female calf by FACS was not yet extensively completed (only one experiment was performed). There is some background noise due to Neu5Gc remnants of the culture conditions; on the other end, also the male cells used as negative control that was completely clear in a previous experiment (Figure 3B) had the same right shift for Neu5Gc (Figure 4B). We can conclude that the generation of DKO cattle is possible using the latest genome editing technologies combined with SCNT. This will offer the opportunity to use novel biological materials of bovine origin for medical and industrial application as well as for human consumption in the form of beef or dairy products for allergic individuals.

## CONFLICT OF INTERESTS

The authors declare no conflict of interests.

## AUTHOR CONTRIBUTION

AP, EC, JPS and CG conceived the project and designed the experiments. AP, IL, RD, EZ, GL, JPJ and SC performed the experiments, collected and analysed the data. JMB, EC, JPS, VPK, CC, GG, MG and TB provided advice for experimental design discussion of results and review of the manuscript. EC, JPS and CG secured the funding. CG, AP and IL wrote the manuscript. All authors reviewed and approved the manuscript.
