# Supplementary material for: Generation of cattle knockout for galactose‐α1,3‐galactose and N‐glycolylneuraminic acid antigens
Source: Xenotransplantation. 2019 May 22;26(5):e12524. doi: 10.1111/xen.12524 (PMC6852128; doi:10.1111/xen.12524)
Supplement: Supplementary file 1 [file XEN-26-na-s001.docx]

**Supplementary information**

**Supp. Table 1.** *Indels* characterizing the edited bovine fibroblast colonies (male and female selected for the SCNT) and the cloned calves.

| **Colony ID** | **Sex** | ***GGTA1*** | ***CMAH*** | **Calf ID** |
| --- | --- | --- | --- | --- |
| A4 | Male | **I allele**  del AGACCCTGGGCGAGTCGG  TGG (21 bp)  **II allele**  del GTGTTTAAGATCAAGCCTG  AGAAGAGGTGGCAGGACATCAGCATGATGCGCATGAAGACTATCGGGGAGCACATTGTGGCCCACATCCAGCATGAGGTTGACTTCCTTTTCTGCATGGATGTGGACCAGGTCTTCCAAGACAAGTTTGGGGTGGAGACCCTGGGCGAGTCG (del 171 bp) | del GGCAGGCAAGTGAG  GGA (17 bp) | 9161 |
| E3 | Male | del AGTCGGTG (8 bp) | **I allele**  Substitution: ATG (START)  to TAA (STOP)  **II allele**  del AGGCAAGTGAGGG (13 bp) | 9162 |
|  |  |  |  |  |
| A6 | Female | del TCTATTTCCCCCCCTCTTCT  TTTCTTTTCCCAGGAGAAAATAATGAATGTCAAA (54 bp) | Substitution: ATG (START) to TAA (STOP) | 9163 |
